# An outbreak of Leptospirosis among United States military personnel in Guam

**DOI:** 10.1186/s40794-017-0059-8

**Published:** 2017-10-30

**Authors:** Alyson J. Brinker, David L. Blazes

**Affiliations:** 1 Guam Naval Hospital, Yigo, Guam; 2 U.S. Navy Hospital Guam, PSC 490 Box 7606 FPO, AP 96538-1600, Tutuhan, Guam; 3 Bill and Melinda Gates Foundation, Seattle, WA USA

**Keywords:** Infectious disease, Leptospirosis, Travel medicine

## Abstract

**Background:**

Leptospirosis is a bacterial zoonotic disease with worldwide distribution.

**Case presentation:**

We describe and discuss the clinical course of a leptospirosis outbreak in a running club called the hash house harriers on Guam.

**Conclusions:**

Leptospirosis is a potentialy life threatening disease, and has had a reemergence given the popularity of travel adventure sports, teams, and clubs around the world. This case presentation highlights the robust prescence of leptospirosis on Guam.

## Background

Leptospirosis is a bacterial zoonotic disease with worldwide distribution and is an important emerging infectious disease [1, 2]. Many wild and domestic animals serve as reservoirs for pathogenic *Leptospira* strains and contaminate the environment by shedding the organisms in their urine. Humans are usually infected through abraded skin or mucous membrane contact with water contaminated by this urine [3, 4]. Athletes, military personnel or others exposed to contaminated water are at risk of contracting leptospirosis. Herein, we report an outbreak of leptospirosis that occurred on the island of Guam, and discuss implications for travelers to tropical regions who may have water exposures.

### Case Presentation

A 24 year old male service member currently stationed on Guam presented with 3 days of subjective fever, headache, myalgias (specifically lower back), severe fatigue, and insomnia that worsened throughout the day. In addition, he noted drenching night sweats and anorexia associated with variable weight loss. He did not report diarrhea, abdominal pain or vomiting. He denied any rashes. He had no ill contacts at the time of initial presentation, and denied recent travel, insect bites, consumption of unusual foods or unpasteurized milk products. He had been immunized against hepatitis A and B, typhoid, influenza, and took no routine medications.

**Fig. 1:**
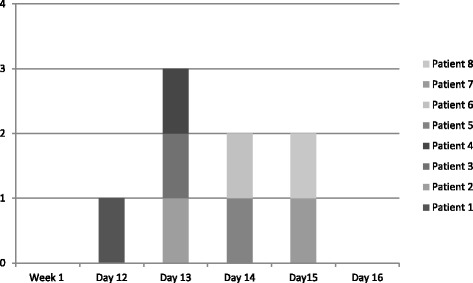
Cases of Leptospirosis by date of onset, Guam

One day after this patient presented, three other patients presented with almost identical symptoms specifically complaining of the headache, extreme fatigue and lower back pain. By day three, four additional patients had presented; two to the clinic, and two to the local ER - Fig. 1, epidemic curve.

All patients denied recent travel, sick contacts or consumption of unpasteurized or raw food. A point source outbreak was suspected because of the epidemic curve. The cluster of cases presented approximately 2 weeks after a particular water exposure during a hash running event on the south side of Guam where 20 or so hikers had gotten lost and spent the night in the jungle. They noted multiple exposures to wet river banks, mud, and swamps.

On exam, all the patients were ill appearing with most prostrate on the examination table. The majority of their exams were normal, with no hepatosplenomegaly, rashes or conjunctival injection noted. Most patients did have abrasions along either the upper arms, or lower legs. All patients demonstrated a temperatures ranging from 101.2–103 F and all were tachycardia.

The majority of laboratory testing was normal, with the exception of slightly elevated serum creatinine and one patient with elevated liver enzymes and creatinine kinase levels. See Table 1.

**Table 1:** Lab results at presentation

Case	Age	Sex	WBC (x10^3^McL)	H/H	Platelets (x10^3^McL)	Creatinine (mg/dL)	AST/ALT (U/L)	Sodium (mmol/L)	Chloride (mmol/L)	Bilirubin (mg/dL)	CK MB(U/L)
1	25	M	4.9	13.8/40.8	139	1.6	37/29	134	97	1.22	8.0
2	24	F	7.0	14/41.1	141	1.0	34/26	131	94	0.38	–
3	21	F	3.4	12.4/36.5	103	1.3	45/44	135	100	0.46	–
4	31	M	9.4	14.8/43.1	145	1.2	40/26	130	94	0.60	–
5	21	M	8.5	14.7/42.4	155	1.3	203/890	135	95	0.84	756.0
6	26	M	5.37	14.8/43.0	235	1.3	28/26	139	98	1.6	–
7	22	M	4.2	14.0/41.0	153	1.4	29/26	131	94	1.2	7.0
8	34	M	4.7	13.9/40.0	123	1.2	37/39	139	93	0.5	–

Microbiologic testing included negative blood culture and multiple serologic studies, the results of which are shown in Table 2. Of note, all patients demonstrated a seroconversion to leptospirosis on convalescent blood samples. Clinical course: All patients responded well to treatment with intravenous normal saline and antiemetics. All were empirically treated with doxycycline 100 mg twice daily and their symptoms resolved over the course of 3 to 4 days. Anorexia continued well into the third day of treatment with antibiotics, with most reporting weight loss between 5 and 15 lb.

**Table 2:** Serologic testing *R: Reactive, NR: Non-reactive, E: Equivocal

Case	Age	Sex	Initial Leptospira IgM	Leptospira IgM (30 days after initial)	EBV/CMV IgM/IgG	Rickettsia panel IgM/IgG(rickettsia rickettsia, Rickettsia typhi)	Dengue IgM/IgG	Hepatitis A IgM	Rapid Flu
1	25	M	NR	R	NR	NR	NR	NR	NR
2	24	F	NR	R	NR	NR	E	NR	NR
3	21	F	NR	R	NR	NR	NR	NR	NR
4	31	M	NR	R	NR	NR	NR	NR	NR
5	21	M	NR	R	NR	E	NR	NR	NR
6	26	M	NR	R	NR	NR	NR	NR	NR
7	22	M	NR	R	NR	NR	NR	NR	NR
8	34	M	NR	R	NR	NR	NR	NR	NR

## Discussion

All patients presented with a clinical syndrome consistent with a broad range of tropical illnesses, see Table 3. Leptospirosis was considered because of the common source of exposure of prolonged contact with contaminated ground water with multiple exposures to rivers, and muddy trails after spending an evening in the southern jungles of Guam near Mount Lam Lam. The initial labs were negative for leptospirosis, but convalescent titers revealed 100% had seroconverted. Due to the remote location of the health care facility, confirmatory microagglutination testing (MAT) was not able to be completed.

**Table 3:** Differential Diagnosis

Dengue	
Chikungunya	
Enterovirus	
Hantavirus	
Hepatitis A	
Rickettsial infection	
Brucellosis	
Malaria	
Meningitis	
Q Fever	
Rickettsial infection	
Viral hemorrhagic fever	
Measles	
Rubella	
Mononucleosis	

Epidemiologically, it is of interest to note a progressive number of outbreaks associated with the increasingly popular sports of international adventuring racing. A 2000 Eco-Challenge in Borneo reports that at least 25% of the participants developed leptospirosis after returning home. Additional outbreaks of leptospirosis have also been investigated in a Triathlon in Springfield, Illinois in 1998, a multisport race in Florida, an endurance jungle race in Martinique, and a triathlon in Langau, Austria, with 15, 12, 9, and 4 confirmed cases respectively [5–9]. Ecotourism, and the popularity of such jungle athletic racing groups and clubs like hashing risk exposing participants to a wide range of pathogens, some of which are only tropical, but also includes pathogens such as leptospirosis, and rickettsia with worldwide distributions. This reinforces the importance of asking a travel history in every patient with a fever, and always practicing engaged preventative medicine by keeping travelers and athletes aware of these bacteria, viruses, and the risks of transmission, and educated about the appropriate barrier, and chemoprophylaxis.

Several studies suggest that doxycycline can be used either as a chemoprophylaxis or as a post exposure prophylaxis and empiric treatment, however the evidence of effectivenss is limited. [10–12]. A study of U.S. Army soldiers participating in a training exercise in Panama, found efficacy of doxycycline administered 200 mg once daily [13]. This case particularly demonstrates that active duty military personnel who travel extensively for their careers, and recreation, also frequently participate in field exercises and tend to be drawn to the active competitive adventure races which can expose them, and heavily impact military readiness encouraging an astute clinician to also always inquire about occupational status.

### Cultural context (text box)

Hash House Harriers is a proverbial “drinking club with a running problem” with organized chapters throughout the world [14]. On weekends, the local chapter on Guam frequently explored the jungle with runs over 4–6 miles of remote terrain including rivers, waterfalls, lakes, and muddy swamps.

The chammorrians (the locals) blamed this outbreak on the tutamo’nas. (people before recorded time), the ghostly apparitions of the ancient people of Guam. The taotaomo’nas of Guahan are said to roam the jungles and are present around the ancient latte ruins, large basalt and coral boulders and caves, as well as amongst the thick dense hanging roots of the Banyan Trees. Legend has it that if one enter the jungles and disturbs the taotaomo’nas, particularly around sunset, they may pinch you, leaving red marks or swellings on your body, or they may cause illnesses which are difficult to diagnose by conventional doctors, such as leptospirosis. [13, 15]

## Conclusions

Leptospirosis is important reemerging tropical disease, especially in an area with an extensive military population. It highlights the existence of the disease on the island of Guam, and also demonstrates the developing association of leptospirosis case outbreaks among world traveling adventure racers in sports such as hashing. This manuscript is important for epidemiological reasons and to discourse the risk factors, and the clinical signs and symptoms of leptospirosis in Guam.
